# Mediation analysis of the relationship between institutional research activity and patient survival

**DOI:** 10.1186/1471-2288-14-9

**Published:** 2014-01-22

**Authors:** Justine Rochon, Andreas du Bois, Theis Lange

**Affiliations:** 1 Institute of Medical Biometry and Informatics, University of Heidelberg, Im Neuenheimer Feld 305, Heidelberg 69120, Germany; 2 Department of Gynecology and Gynecologic Oncology, Kliniken Essen-Mitte, Essen, Germany; 3 Department of Biostatistics, University of Copenhagen, Copenhagen, Denmark

**Keywords:** Trial effect, Research activity, Healthcare outcomes, Mediation, Survival analysis

## Abstract

**Background:**

Recent studies have suggested that patients treated in research-active institutions have better outcomes than patients treated in research-inactive institutions. However, little attention has been paid to explaining such effects, probably because techniques for mediation analysis existing so far have not been applicable to survival data.

**Methods:**

We investigated the underlying mechanisms using a recently developed method for mediation analysis of survival data. Our analysis of the effect of research activity on patient survival was based on 352 patients who had been diagnosed with advanced ovarian cancer at 149 hospitals in 2001. All hospitals took part in a quality assurance program of the German Cancer Society. Patient outcomes were compared between hospitals participating in clinical trials and non-trial hospitals. Surgical outcome and chemotherapy selection were explored as potential mediators of the effect of hospital research activity on patient survival.

**Results:**

The 219 patients treated in hospitals participating in clinical trials had more complete surgical debulking, were more likely to receive the recommended platinum-taxane combination, and had better survival than the 133 patients treated in non-trial hospitals. Taking into account baseline confounders, the overall adjusted hazard ratio of death was 0.58 (95% confidence interval: 0.42 to 0.79). This effect was decomposed into a direct effect of research activity of 0.67 and two indirect effects of 0.93 each mediated through either optimal surgery or chemotherapy. Taken together, about 26% of the beneficial effect of research activity was mediated through the proposed pathways.

**Conclusions:**

Mediation analysis allows proceeding from the question “Does it work?” to the question “How does it work?” In particular, we have shown that the research activity of a hospital contributes to superior patient survival through better use of surgery and chemotherapy. This methodology may be applied to analyze direct and indirect natural effects for almost any combination of variable types.

## Background

Well-conducted trials are critical to advancements in oncology. However, such trials require patients and healthcare providers who are willing to participate in clinical research. The effect of participation in clinical trials is controversially discussed in the literature. Reviews on this effect have been published in the past, but these have usually focused on comparisons between ‘trial patients’ and ‘non-trial patients’ and authors have come to conflicting conclusions [1-3].

In the last years another relevant aspect of participation in clinical trials has emerged: “Do healthcare institutions or service providers who are active in research deliver better care and outcomes than those who do not participate in clinical research?” [4], p. 6]. According to Pater et al. [5], especially from the healthcare policy perspective, this question should be the main focus of future research because benefit from a research-active institution will affect many more patients than just the small proportion of participants in clinical trials. In addition, this issue may also be of greater importance to patients who have to decide where to go to receive healthcare and by whom. However, only one review conducted by Clarke and Loudon [6] included a literature search to identify studies addressing the relationship between institutional research activity and patient outcomes. The available evidence suggests that patients benefit from being treated by practitioners or in institutions participating in trials. The authors concluded that research activity might result in better health care outcomes, although the reason for this effect remains unclear. Indeed, most studies focus on answering the pragmatic question of whether research activity has an effect on patient outcome. However, little attention is being paid to the explanation of such effects (“How does it work?”, i.e. the assessment of mediation).

**Figure 1:**
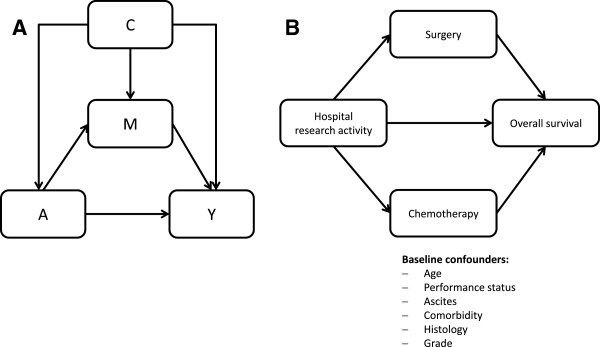
**Path diagrams. (A)** Path diagram relating exposure A to a mediator M and an outcome Y in presence of known baseline confounders C. **(B)** Path diagram relating hospital research activity to two mediators and overall survival time in presence of known baseline confounders.

Since the publication of the seminal paper by Baron and Kenny [7], testing for mediation has been an integral part of statistical analysis in psychology. In epidemiology, both practical and theoretical aspects of mediation have been considered since Robins and Greenland [8]. The importance of mediation is well argued in Hafeman and Schwartz [9] where they demanded the opening of the ‘black box’ when investigating exposure-disease relationships. The core element of mediation analysis is the estimation of two effects: The natural direct effect of exposure on outcome and the natural indirect effect acting through a so-called mediator that is assumed to reflect a specific causal pathway between exposure and outcome. The total effect is the aggregate of these two effects (Figure 1A). Specifically, the natural direct effect is the effect one would observe if the exposure could be changed without inducing a change in the mediator. The natural indirect effect is the effect one would observe if the mediator could be changed as it would when the exposure was manipulated (without actually changing the exposure).

For normally distributed mediators and outcomes and in the absence of interactions or non-linear effects, natural direct and indirect effects can be estimated by a stepwise approach based on standard linear regressions [7]. In short, models are fitted for the outcome both with and without the mediator, and the difference in the coefficients for the exposure is taken as a measure of the indirect or mediated effect. In oncology, however, the outcome of interest is often patient survival. Survival times are known to be non-normally distributed and typically right censored. Therefore, existing techniques for mediation analysis are not applicable to survival data, and several authors have shown that the linear regression approach cannot be simply translated to logistic regression or proportional hazard models [10-12].

The aim of this article is to introduce a recently developed methodology that can be used to assess mediation on all outcome types including survival data, and to use this method to explain the effects of institutional research activity on ovarian cancer patient survival.

## Methods

### Patients

The study is part of an ongoing quality assurance program (QS-OVAR) initiated in 1999 by the Arbeitsgemeinschaft Gynaekologische Onkologie (AGO) Organkommission OVAR, a subcommittee of the German Cancer Society. The aim of this program is to describe the pattern and quality of care of patients with ovarian cancer in Germany as well as to improve their outcome. The hypothesis is that research-active hospitals yield improved outcomes for their patients. Research activity at hospital level is defined as hospital participation in prospective clinical trials conducted by one of the two German cooperative study groups, the AGO Study Group Ovarian Cancer (AGO-OVAR) and the Northeastern Society of Gynecologic Oncology (NOGGO). More specifically, all patients treated in hospitals participating in clinical trials are compared with all patients treated in hospitals that did not participate in any clinical trials of the AGO-OVAR and NOGGO.

The mediation analysis of the effect of research activity on healthcare outcomes is illustrated through QS-OVAR 2001. This study involved 165 hospitals and 476 patients with early and advanced ovarian cancer diagnosed in the third quarter of 2001. The primary outcome measure was overall survival. Secondary outcomes were the receipt of standard care with regard to surgery and chemotherapy. Details on study design and results have been described elsewhere [13,14]. Here, we will focus on patients with advanced ovarian cancer because most death events in QS-OVAR 2001 occurred in patients with advanced stages Fédération Internationale de Gynécologie et d'Obstétrique (FIGO) IIB–IV, whereas only a few events were observed in FIGO I–IIA patients. Furthermore, treatment options differ markedly between early and advanced stage disease. According to the German guidelines, patients with ovarian cancer FIGO IIB and higher should receive surgery including maximal debulking as well as chemotherapy with carboplatin and paclitaxel [15]. We thus considered two potential mediators. First, ‘optimal debulking’ with postoperative tumor residuals up to 1 cm was regarded as standard care with respect to surgery (i.e. ‘optimal surgery’). Second, each platinum-taxane combination was considered adherent to treatment guidelines regarding chemotherapy (i.e. ‘optimal chemotherapy’).

The use of data included in this study was approved by the AGO-OVAR and the AGO Ovarian Committee. All data was anonymized prior to analysis. The study was approved by the Ethics Committee of the Medical Faculty of the University of Heidelberg, Germany (study number: S-446/2013).

### Statistical analyses

All analyses were conducted in R version 3.0.2 [16]. Continuous data were summarized with median and interquartile range (IQR), categorical data by counts and percentages. Survival curves were generated by the Kaplan-Meier method. The Cox proportional hazards model with robust variance estimator was used to assess the relationship between hospital participation in clinical trials and overall survival. Logistic regression models were fitted for ‘optimal surgery’ and ‘optimal chemotherapy’ with generalized estimating equations to account for clustering of patients within hospitals. The following patient and disease characteristics were assumed to control for confounding: age at diagnosis (continuous, in 5 years units), Eastern Cooperative Oncology Group (ECOG) performance status (>1 vs. 0/1), ascites (> 500 ml vs. ≤ 500 ml), comorbidity (present vs. none), histology (serous vs. other), and grade (G 3/4 vs. G 1/2).

Mediation analysis was performed using the approach proposed by Lange et al. [17]. This approach is based on the counterfactual framework [18] and allows decomposition of the total effect of a given exposure *A* on the outcome *Y* into a natural direct effect (*A* → *Y* in Figure 1A) and a natural indirect effect through a mediator *M* (*A* → *M* → *Y* in Figure 1A). In case of a time-to-event outcome *Y*, a binary exposure *A*, a binary mediator *M* and a number of baseline confounders *C*, Lange et al. showed that unbiased estimates for the direct and indirect effect are obtained from weighted Cox regression of the time-to-event outcome on *A*, A* and *C* using a duplicated data set. In the first replication A* takes the original value of the exposure. In the second replication A* takes the opposite (‘counterfactual’) value of the exposure. The weights are determined by *W*^*c*^ = *P*(*M* | A*, *C*) / *P*(*M* | *A*, *C*), with *P*( · ) deriving from a logistic regression of the mediator *M* on the exposure and the baseline confounders [17, Appx. 4]. Assuming non-informative censoring and proportional hazards, the weighted Cox model then yields hazard ratios for *A* and A* that serve as estimates for the natural direct effect and indirect effect, respectively. The product of the two hazard ratios yields the hazard ratio for the total effect. Standard errors and confidence intervals can, for example, be determined by bootstrap methods.

In our study, we explored two binary mediators (surgery and chemotherapy) of the effect of hospital research activity on survival (Figure 1B). Under the assumption of separate causal pathways through the two mediators, unbiased point estimates for the natural direct effect and the natural indirect effects related to the two mediators were obtained by a weighted Cox regression of the outcome on the exposure, the baseline confounders and two additional counterfactual variables *A*_1_* and *A*_2_* that were systematically manipulated in four replicates of the original data [19]. Confidence intervals for mediation effects that account for clustering of patients within hospitals were obtained using simple random cluster sampling and 10,000 bootstrap simulations. In all analyses, results were considered statistically significant if the 95% confidence interval (CI) for the hazard ratio (HR) or odds ratio (OR) did not include 1.

In the additional material (see Additional file 1), we have provided a detailed description of the analysis with the corresponding R code to enable future researchers to assess mediation in a survival context. There, we also show how we tested for interactions between exposure and confounders, how we assessed the linearity assumption for all variables, and how we allowed for misspecification in the mediators. Finally, this description includes sensitivity analyses with only one mediator representing adherence to treatment guidelines with regard to surgery and chemotherapy as a binary and an ordinal variable.

## Results

Altogether, two-thirds of the 476 patients diagnosed in the third quarter of 2001 had advanced ovarian cancer. The 352 patients with FIGO IIB or higher were treated in 149 hospitals. The number of patients per hospital ranged from 1 to 12, the median number was two. 219 patients (62%) were treated in 77 hospitals participating in clinical trials, and 133 patients (38%) were treated in 72 non-trial hospitals. However, only 59 (27%) of the 219 patients in trial hospitals were actually enrolled in clinical trials which included a trial that did not show any superiority of the experimental treatment [20]. Patient and disease characteristics at diagnosis for both groups are listed in Table 1.

**Table 1:** Patient and disease characteristics at diagnosis

	**Trial hospitals**		**Non-trial hospitals**	
No. of patients, *N* (%)	219	(62.2)	133	(37.8)
Age (years)				
Median (IQR)	64	(57–73)	66	(56–74)
Performance status, *N* (%)				
ECOG 0/1	166	(75.8)	101	(75.9)
ECOG > 1	53	(24.2)	32	(24.1)
Ascites, *N* (%)				
≤ 500 ml	113	(51.6)	59	(44.4)
> 500 ml	106	(48.4)	74	(55.6)
Comorbidity, *N* (%)				
None	166	(75.8)	91	(68.4)
Present	53	(24.2)	42	(31.6)
Histology, *N* (%)				
Serous	167	(76.3)	96	(72.2)
Other	52	(23.7)	37	(27.8)
Grade, *N* (%)				
G 1/2	97	(44.3)	69	(51.9)
G 3/4	122	(55.7)	64	(48.1)

**Figure 2:**
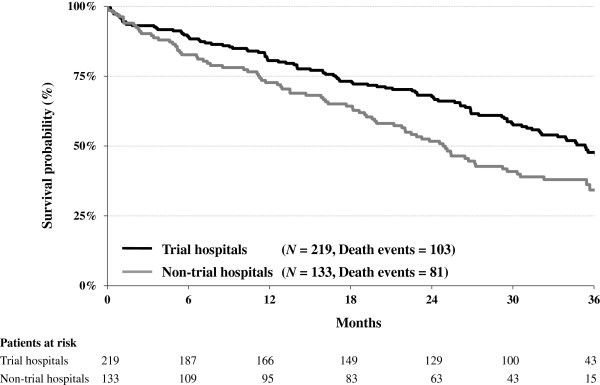
Kaplan-Meier survival curves in advanced ovarian cancer according to hospital participation in clinical trials.

During the follow-up period of three years, 184 out of 352 patients with advanced ovarian cancer died. The median overall survival time was 35 months for the 219 patients treated in hospitals participating in clinical trials compared to 25 months for the 133 patients treated in non-trial hospitals (Figure 2). This survival benefit remained stable even after adjustment for all patient and disease characteristics listed in Table 1, as well as after adjustment for clustering of patients within hospitals in a multivariable Cox model. The total effect of research activity (adjusted for known confounders) can be found in Table 2: The adjusted HR of death was 0.58 (95% CI: 0.42 to 0.79).

**Table 2:** Multivariable Cox regression analysis for overall survival in patients with advanced ovarian cancer

**Variable**	***N***	**Events**	**HR**	**95% CI**
Research activity				
Non-trial hospital	133	81	1	Reference
Trial hospital	219	103	0.58	[0.42; 0.79]
Age				
Continuous (5 years)	352	184	1.24	[1.14; 1.34]
Performance status				
ECOG 0/1	267	118	1	Reference
ECOG > 1	85	66	2.02	[1.38; 2.96]
Ascites				
≤ 500 ml	172	74	1	Reference
> 500 ml	180	110	1.77	[1.23; 2.53]
Comorbidity				
None	257	116	1	Reference
Present	95	68	1.46	[1.08; 1.97]
Histology				
Other	89	40	1	Reference
Serous	263	144	1.29	[0.85; 1.94]
Grade				
G 1/2	166	81	1	Reference
G 3/4	186	103	1.10	[0.80; 1.50]

**Figure 3:**
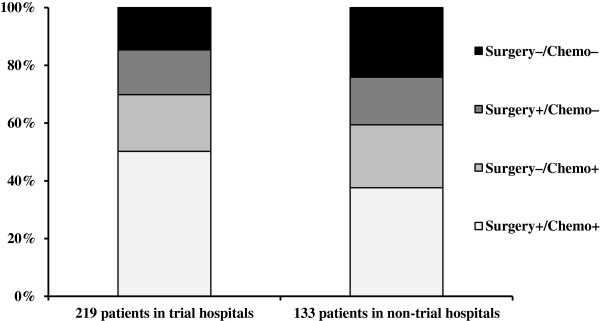
Adherence to treatment guidelines with regard to surgery and chemotherapy in advanced ovarian cancer according to hospital participation in clinical trials.

How much of the total beneficial effect of research activity is mediated through optimal debulking and standard chemotherapy? In the first step, we separately explored the relationship between hospital participation in clinical trials and the two independent mediators. The proportion of patients receiving the recommended platinum-taxane combination was 70% in research-active hospitals and 59% in non-trial hospitals (adjusted OR = 1.64, 95% CI: 0.91 to 2.95). 66% of the 219 patients treated in research-active hospitals had maximal tumor residuals of 1 cm in contrast to 54% of the 133 patients treated in non-trial hospitals (adjusted OR = 1.57, 95% CI: 0.92 to 2.69). Altogether, about 50% of patients in research-active hospitals and 38% of patients in non-trial hospitals received the combination of optimal debulking with standard chemotherapy (Figure 3).

In the next step, we explored the effect of standard care on survival, again by means of a multivariable Cox model. As expected, both potential mediators were associated with survival. The adjusted HR of death for optimally debulked patients was 0.46 (95% CI: 0.34 to 0.63) compared to patients with a postoperative tumor residual larger than 1 cm. Similarly, we found a survival benefit for patients treated with platinum-taxane chemotherapy (adjusted HR = 0.42, 95% CI: 0.28 to 0.62).

Finally, we used the approach of Lange et al. [17] to test if the observed positive effect of institutional research activity on patient survival is at least partially mediated through surgery and chemotherapy (Figure 1B).

The effects of hospital participation on overall survival in terms of natural direct and indirect hazard ratios can be summarized as follows (Table 3): The total HR of 0.58 was decomposed into a direct HR of research-activity of 0.67 (95% CI: 0.47 to 0.92) and an indirect HR for both mediators of 0.87 (95% CI: 0.75 to 0.98). The indirect HR with regard to surgery alone was 0.93, the corresponding indirect HR with respect to chemotherapy was also 0.93; thus the resulting total effect was 0.67 × 0.93 × 0.93 = 0.58. The proportion mediated through surgery and chemotherapy was similar and was about 13% for each mediator on the log HR scale. Altogether, about 26% of the total effect of hospital research activity on survival was mediated through the proposed pathways (95% CI: 3% to 69%).

**Table 3:** Mediation analysis: Total, direct and indirect effects with 95% confidence intervals

**Effect (of trial hospital vs. non-trial hospital)**	**HR**	**95% CI**
Total effect	0.58	[0.41; 0.80]
Direct effect	0.67	[0.47; 0.92]
Indirect effect (through surgery)	0.93	[0.84; 1.02]
Indirect effect (through chemotherapy)	0.93	[0.84; 1.01]
Indirect effect (through both surgery and chemotherapy)	0.87	[0.75; 0.98]

## Discussion

The effect of institutional research activity on patient outcomes has not yet been investigated extensively, despite its great relevance to healthcare providers, policy makers, and patients. So far, only a few studies have examined the association between patient outcomes and institutional participation in clinical trials, as opposed to trial participation of individual patients [6]. Thus, the authors of the 2011 special issue of Annals of Oncology entitled “Clinical Research and Healthcare Outcomes: A Workshop at the International Agency for Research on Cancer, Lyon” agreed that further research on this topic is urgently required. If research activity has beneficial effects on patient outcomes, these benefits are not solely restricted to research participants.

Investigating the relationship between research activity at the level of institutions or providers and outcomes at the level of patients is not as easy as it seems at first glance. Researchers interested in this relationship are confronted with several practical challenges, such as getting data from less research-active institutions, as well as with several methodological challenges, for instance, the choice of the appropriate study design [5]. In addition, sole focus on establishing effects and measuring the potential benefits of research activity on healthcare outcomes is often considered insufficient [6]. The assessment of variables mediating the effects may be helpful in explaining the effects of exposure or in investigating the reasons why an exposure failed to yield an expected outcome. More importantly, knowledge of such mechanisms enables specific measures to improve healthcare at the individual level and at the institutional level—and such measures may be implemented even in the absence of exposure. Krzyzanowska et al. [21] described a conceptual framework for understanding how institutional research activity might lead to better outcomes, even for patients who are not participants in a research project. The authors pointed out that the processes of care received by patients may have a strong impact on outcomes and that such processes may systematically differ between research-active and research-inactive settings. For example, institutions that actively participate in research may be more likely to follow clinical guidelines for cancer treatment. In addition, participation in research may facilitate early access to new treatment approaches, allowing the faster implementation of new evidence into practice.

The main purpose of the present work was to explore the mechanisms underlying the association between institutional research activity and patient outcomes. We first re-examined the association between institutional research activity and survival described by du Bois et al. [13]. Using the data of 352 patients with advanced ovarian cancer, we showed that hospital participation in clinical trials was associated with improved survival. The effect observed in our study was large and clearly relevant because of a relative reduction in the risk of death by 42% in favor of research activity. Whether this effect is in line with the literature is difficult to answer because, with respect to ovarian cancer, trial participation has hardly been investigated outside Germany. Furthermore, only a few studies have examined the association between patient outcomes and research activity at the level of health care institutions [6].

As expected, patients with ‘optimal surgery’ and ‘optimal chemotherapy’ lived longer than patients who were not treated according to the standard of care. In addition, patients treated in trial hospitals were more likely to receive optimal treatment than patients treated in non-trial hospitals. We thus confronted the question of how much of the effect of hospital participation in clinical trials on patient survival was mediated through optimal debulking and optimal chemotherapy selection. To answer this question, we used a recently developed methodology for assessing mediation in the context of a survival analysis [17]. Taking into account several known baseline confounders, the overall hazard ratio (total effect) of 0.58 was decomposed into a direct effect of research activity of 0.67 and two indirect effects of 0.93 each mediated through surgery and chemotherapy. The aggregate indirect effect through both mediators was 0.87, that is, about 26% of the beneficial effect of research activity was mediated through both surgery and chemotherapy. In conclusion, trial participation of a hospital contributed at least partially to a superior outcome through the better quality of treatment provided.

The probability of surviving ovarian cancer depends on (1) patient characteristics (e.g. age), (2) tumor biology (e.g. stage), and (3) the quality of treatment (e.g. surgical outcome, selection of chemotherapy regimen). The first two factors are hard to change but the quality of treatment is susceptible to direct influence and thus seems to be of utmost relevance when considering efforts to improve the outcome of this disease. The implementation of standards into clinical routine in our study was not satisfactory and still needs improvement; however, taken both mediators together, treatment standards were more strictly implemented in trial hospitals than in non-trial hospitals. The good news is that clinicians can influence the implementation of standards, regardless of whether they are employed in research-active hospitals or not.

The main limitation of our study is the lack of randomization of patients into research-active and research-inactive hospitals. Such a randomized controlled trial would facilitate a clear causal interpretation but would also be hard to implement. When randomization is not possible, well-conducted observational studies can provide the necessary data to guide the future development of clinical research and healthcare. In particular, mediation analysis can help explore the mechanisms by which research activity leads to the outcome of interest. The key assumption for mediation analysis is that all relevant confounders are included into the analysis. To be more precise, the approach proposed by Lange et al. [17] requires that the considered variables are sufficient for controlling the confounding of 1) the exposure-outcome relation, 2) the exposure-mediator relation, and 3) the mediator-outcome relation. We addressed this issue by incorporating all established prognostic factors into the models for the mediator and the outcome. In particular, age at diagnosis, FIGO stage, ECOG performance status, volume of ascites, histology, grade, and comorbidity are well-known prognostic factors for survival in ovarian cancer [22-24]. These factors also influence treatment recommendations.

There must be no other variables (measured or unmeasured) that confound the mediator-outcome relation which are themselves affected by the exposure; this last assumption essentially ensures that each of the considered pathways between exposure and outcome does indeed represent a unique causal mechanism. In observational studies, these assumptions are inherently untestable and must instead be justified by means of knowledge about the biological processes under consideration. In our study, for example, we did not collect information on socioeconomic factors (e.g. income or insurance status). Patients with a higher socioeconomic status (SES) may find it easier and hence choose to travel to specialized or better rated hospitals or institutions that are research-active and participate in clinical trials. In contrast, cancer patients with a low SES may be less likely to choose research-active hospitals because of the lack of corresponding health care information. The total effect of research activity would then at least be partially due to better general survival rates of patients with a high SES. Lower socioeconomic status has occasionally been associated with lower likelihood of receiving surgery and chemotherapy but does not seem to be an independent prognostic factor for survival in ovarian cancer [25,26].

Finally, the mediation analysis is only valid if the logistic regressions are adequate descriptions of the mediators. Sensitivity analyses showed that misclassification of the mediator will bias the estimates of the indirect effect towards one and the direct effect away from one. In our study, measurement error was minimized by objectively assessing the quality of chemotherapy and by evaluating surgical outcome (i.e. tumor residual) in a standardized manner.

## Conclusions

Mediation analysis, which is regularly and successfully applied elsewhere in health care, has a great potential to be used as an instrument for investigating underlying mechanisms. This tool allows analysis to go beyond the question “Does it work?” In particular, mediation analysis has shown that the research activity of a hospital contributes to superior patient survival through better adherence to treatment guidelines with regard to surgical outcome and selection of chemotherapy. Yet, the analysis of the ovarian cancer study presented in this article demonstrates only one way of applying this methodology, but the potential for this technique is much wider. This methodology, which can be conducted with standard software, may be applied to analyze direct and indirect effects for almost any combination of variable types (in particular, the mediator and outcome can be binary, ordinal, categorical, or continuous, and the outcome can also be survival time). Researchers interested in the question “How does it work?” are thus strongly encouraged to use mediation analysis in their investigations.

## Competing interests

The authors declare that they have no competing interests.

## Authors’ contributions

JR and AdB jointly designed the study. JR and TL carried out the analysis. JR drafted the manuscript. All authors contributed to revisions of the manuscript, and read and approved the final version.

## Pre-publication history

The pre-publication history for this paper can be accessed here:

http://www.biomedcentral.com/1471-2288/14/9/prepub

## Supplementary Material

Additional file 1 A detailed description of the mediation analysis with the corresponding R code. Click here for file
