# Supplementary material for: Mediation analysis of the relationship between institutional research activity and patient survival
Source: BMC Med Res Methodol. 2014 Jan 22;14:9. doi: 10.1186/1471-2288-14-9 (PMC3917547; doi:10.1186/1471-2288-14-9)
Supplement: Additional file 1 — A detailed description of the mediation analysis with the corresponding R code. [file 1471-2288-14-9-S1.pdf]

Mediation analysis of the relationship between institutional research activity and patient survival

Data description

This additional material describes the necessary steps for the mediation analysis of patient survival in hospitals participating in the QS-OVAR 2001 quality assurance program. Details on study design and results have been described elsewhere [1, 2]. Here, we focus on patients with advanced ovarian cancer. Patient outcomes are compared between hospitals participating in clinical trials and non-trial hospitals. Surgical outcome and chemotherapy selection are explored as potential mediators of the effect of hospital research activity on patient survival. The analysis is written in R statistical programming language [3] version 3.0.2. The data set includes the following variables:

- HospID: Hospital identifier
- Trial: Research activity (Boolean, TRUE if hospital participates in clinical trials)
- Age5: Age of patient at diagnosis (in units of 5 years)
- ECOG: ECOG performance status (0/1 vs. > 1)
- Ascit: Ascites (≤ 500 ml vs. > 500 ml)
- Comorb: Comorbidities (Boolean, TRUE if comorbidities present)
- Histo: Tumor histology (Serous vs. Other)
- Grade: Tumor grade (Grade 1/2 vs. Grade 3/4)
- Chem: Optimal chemotherapy (Boolean, TRUE if platinum-taxane combination)
- Surg: Optimal surgery (Boolean, TRUE if tumor residual ≤ 1 cm)
- OS: Overall survival (months)
- Status: Censoring indicator (Boolean, TRUE if patient died)

Assuming that the data is stored in the standard comma-separated values format, it is stored in a dataframe `d` using the following command:

```
d = read.csv('ovar2001.csv')
d = d[order(d$HospID), ] # consecutive rows per cluster for geeglm
d$ECOG = relevel(d$ECOG, ref='0/1')
```

The second command ensures that patient data deriving from the same hospital are stored in consecutive rows of the dataframe. This is required by the command `geeglm` from library geepack (Halekoh et al. [4]) to work properly. The third command defines the reference category for the ECOG performance status.

Total effect

The total effect of research activity on survival is estimated by standard Cox proportional hazards regression (library survival, Therneau [5]). Covariates are included to adjust for the known baseline confounders (Age5, ECOG, Ascit, Comorb, Histo, Grade), and the standard error of the effect estimate is corrected for the clustering of patients within hospitals:

```
library(survival)
TE = coxph(Surv(OS, Status) ~ Trial + Age5 + ECOG + Ascit + Comorb +
    Histo + Grade + cluster(HospID), data=d)
summary(TE)
```

The last command produces the following output:

```
n = 352, number of events = 184
                coef exp(coef) se(coef) robust se      z Pr(>|z|)
TrialTRUE    -0.5486    0.5778   0.1534    0.1573 -3.49  0.00049 ***
Age5          0.2113    1.2352   0.0420    0.0411  5.14  2.8e-07 ***
ECOG>1        0.7023    2.0183   0.1836    0.1947  3.61  0.00031 ***
Ascit>500 ml  0.5699    1.7682   0.1547    0.1835  3.11  0.00189 **
ComorbTRUE    0.3758    1.4561   0.1692    0.1546  2.43  0.01509 *
HistoSerous   0.2539    1.2890   0.1852    0.2098  1.21  0.22624
GradeG3/4     0.0909    1.0951   0.1506    0.1589  0.57  0.56728
---
Signif. codes:  0 '***' 0.001 '**' 0.01 '*' 0.05 '.' 0.1 ' ' 1

             exp(coef) exp(-coef) lower .95 upper .95
TrialTRUE        0.578      1.731     0.425     0.786
Age5             1.235      0.810     1.140     1.339
ECOG>1           2.018      0.495     1.378     2.956
Ascit>500 ml     1.768      0.566     1.234     2.533
ComorbTRUE       1.456      0.687     1.075     1.972
HistoSerous      1.289      0.776     0.854     1.945
GradeG3/4        1.095      0.913     0.802     1.495

Concordance= 0.732  (se = 0.023 )
Rsquare= 0.292   (max possible= 0.996 )
Likelihood ratio test= 121  on 7 df,   p=0
Wald test            = 132  on 7 df,   p=0
Score (logrank) test = 132  on 7 df,   p=0,   Robust = 58.3  p=3.28e-10
```

The results indicate that research activity of the hospital has a beneficial influence on patient survival (hazard ratio for Trial HR = 0.578, with 95% confidence interval (CI) between 0.425 and 0.786, see columns `exp(coef)`, `lower .95`, and `upper .95`, respectively).

Effect on the mediators

Main question of the present study is whether the beneficial effect of hospital trial participation on patient survival is mediated through better adherence to treatment guidelines with regard to chemotherapy selection and surgical outcome. Therefore, in the next step, we investigate the effect of research activity on the two binary mediators optimal chemotherapy and optimal surgery. Odds ratios for the two mediators are obtained by logistic regression models. Again, covariates are included to adjust for known baseline confounders. Clustering of patients into hospitals is accounted for by means of generalized estimation equations (library geepack, [4]).

```
library(geepack) # Needs to be downloaded from CRAN
MChem = geeglm(Chem ~ Trial + Age5 + ECOG + Ascit + Comorb + Histo + Grade,
    family=binomial(), data=d, id=d$HospID)
summary(MChem)
```

The following output is generated for optimal chemotherapy:

```
               Estimate Std.err  Wald Pr(>|W|)    
(Intercept)      6.0102  1.2448 23.31  1.4e-06 ***
TrialTRUE        0.4948  0.2987  2.74    0.098 .  
Age5            -0.4194  0.0830 25.51  4.4e-07 ***
ECOG>1          -1.3731  0.3409 16.23  5.6e-05 ***
Ascit>500 ml     0.2284  0.2730  0.70    0.403    
ComorbTRUE      -0.5350  0.2729  3.84    0.050 *  
HistoSerous      0.3847  0.3415  1.27    0.260    
GradeG3/4        0.0604  0.2766  0.05    0.827    
---
Signif. codes:  0 '***' 0.001 '**' 0.01 '*' 0.05 '.' 0.1 ' ' 1

Estimated Scale Parameters:
            Estimate Std.err
(Intercept)     1.12   0.889

Correlation: Structure = independence  
Number of clusters:   149  
Maximum cluster size: 12
```

Though not statistically significant at the conventional α = 5%, the results indicate better chemotherapy adherence in the research-active hospitals. The odds ratio is exp(0.495) = 1.64, the 95% confidence limits are obtained by exp(0.495 ± 1.96 × 0.299).

A similar result is obtained for the other mediator, optimal surgery:

```
MSurg = geeglm(Surg ~ Trial + Age5 + ECOG + Ascit + Comorb + Histo +
    Grade, family=binomial(), data=d, id=d$HospID)
summary(MSurg)
```

The following output is generated:

```
               Estimate Std.err  Wald Pr(>|W|)    
(Intercept)      3.4572  0.8602 16.15  5.8e-05 ***
TrialTRUE        0.4535  0.2740  2.74  0.09793 .  
Age5            -0.1718  0.0616  7.78  0.00529 ** 
ECOG>1          -0.9113  0.3370  7.31  0.00685 ** 
Ascit>500 ml    -0.8739  0.2449 12.73  0.00036 ***
ComorbTRUE      -0.4247  0.2652  2.57  0.10923    
HistoSerous     -0.2705  0.3155  0.73  0.39139    
GradeG3/4       -0.0370  0.2736  0.02  0.89247    
```

The odds ratio for optimal surgery amounts to exp(0.454) = 1.57, favoring patients treated in research-active hospitals (not statistically significant, though).

Independence of the two mediators

Analysis of multiple pathways requires mutually independent mediators, conditional on exposure and confounders [6, 7]. Logistic regression of Surgery on Chemotherapy is thus used again, predicting optimal surgery by optimal chemotherapy and controlling for the other confounders.

```
MIndep = geeglm(Surg ~ Chem + Trial + Age5 + ECOG + Ascit + Comorb +
    Histo + Grade, family=binomial(), data=d, id=d$HospID)
summary(MIndep)
```

The non-significant result for Chemo (*P* = 0.685) indicates that the data are at least “consistent” with the assumption of independence (see also the sensitivity analyses):

```
             Estimate Std.err  Wald Pr(>|W|)
(Intercept)    3.2507  1.0021 10.52  0.00118 **
ChemTRUE       0.1312  0.3230  0.17  0.68450
TrialTRUE      0.4454  0.2765  2.59  0.10725
Age5          -0.1624  0.0668  5.91  0.01508 *
ECOG>1        -0.8746  0.3372  6.72  0.00951 **
Ascit>500 ml  -0.8800  0.2501 12.38  0.00043 ***
ComorbTRUE    -0.4115  0.2652  2.41  0.12074
HistoSerous   -0.2773  0.3132  0.78  0.37590
GradeG3/4     -0.0380  0.2733  0.02  0.88937
```

Effect decomposition

The purpose of the mediation analysis is to understand what mediates the effect of research activity on survival. Therefore, the total effect of HR = 0.578 is decomposed into two natural indirect effects mediated by optimal surgery and optimal chemotherapy, as well as a natural direct effect of research activity onto the outcome.

```
doEffectDecomp = function(d)
{
    # Step 1: Replicate exposure variable, predict mediators
    d$TrialTemp = d$Trial
    MChem = glm(Chem ~ TrialTemp + Age5 + ECOG + Ascit + Comorb +
        Histo + Grade, family=binomial(), data=d)
    MSurg = glm(Surg ~ TrialTemp + Age5 + ECOG + Ascit + Comorb +
        Histo + Grade, family=binomial(), data=d)

    # Step 2a: Replicate data with different exposures for chemotherapy
    d1 = d2 = d
    d1$MedChem = d1$Trial
    d2$MedChem = !d2$Trial
    newd = rbind(d1, d2)

    # Step 2b: Replicate data with different exposures for surgery
    d1 = d2 = newd
    d1$MedSurg = d1$Trial
    d2$MedSurg = !d2$Trial
    newd = rbind(d1, d2)

    # Step 3a: Compute weights for chemotherapy
    newd$TrialTemp = newd$Trial
    w = predict(MChem, newdata=newd, type='response')
    direct = ifelse(newd$Chem, w, 1-w)
    newd$TrialTemp = newd$MedChem
    w = predict(MChem, newdata=newd, type='response')
    indirect = ifelse(newd$Chem, w, 1-w)
    newd$WChem = indirect/direct
```

```
    # Step 3b: Compute weights for surgery
    newd$TrialTemp = newd$Trial
    w = predict(MSurg, newdata=newd, type='response')
    direct = ifelse(newd$Surg, w, 1-w)
    newd$TrialTemp = newd$MedSurg
    w = predict(MSurg, newdata=newd, type='response')
    indirect = ifelse(newd$Surg, w, 1-w)
    newd$WSurg = indirect/direct

    # Step 4: Weighted Cox Model
    newd$W = newd$WChem * newd$WSurg
    cox = coxph(Surv(OS, Status) ~ Trial + MedChem + MedSurg + Age5 +
        ECOG + Ascit + Comorb + Histo + Grade, weight=W, data=newd)

    # Return value: Estimates for total, direct, indirect effects
    TE = exp(sum(coef(cox)[c('TrialTRUE', 'MedChemTRUE', 'MedSurgTRUE')]))
    DE = exp(unname(coef(cox)['TrialTRUE']))
    IE = exp(sum(coef(cox)[c('MedChemTRUE', 'MedSurgTRUE')]))
    PM = log(IE) / log(TE)

    return(c(exp(coef(cox)), TE=TE, DE=DE, IE=IE, PM=PM))
}
```

The effect decomposition occurs in several consecutive steps. In the first step, logistic regression is used to get effect estimates for the influence of the exposure (i.e., research activity) on the mediators. In the second step, the dataframe is replicated with different ('counterfactual') values of the exposure. Then weights are determined for the replicated data according to expression W_i^c in Lange et al. ([6], p. 193). The extension of the technique from one to two mediators is described in Lange et al. [7].

The weights are finally used in a Cox model to estimate the hazard ratios related to the direct and the mediated indirect effects. The effect decomposition is packaged in a function `doEffectDecomp` to enable replicated execution for bootstrap confidence intervals. Point estimates of the hazard ratios related to the total, direct and indirect effects are obtained by a direct call of the function:

```
doEffectDecomp(d)
```

These are the point estimates for the different effects and covariates:

```
 TrialTRUE  MedChemTRUE  MedSurgTRUE   Age5  ECOG0/1  Ascit>500 ml
     0.666        0.933        0.930  1.236    2.025         1.783

ComorbTRUE  HistoSerous    GradeG3/4     TE       DE          IE         PM
     1.383        1.276        1.088  0.578    0.666       0.868      0.259
```

Hence, the total HR = 0.578 decomposes into a direct HR = 0.666 and an indirect HR = 0.868. The indirect HR corresponds to the two mediator effects of 0.933 and 0.930 for optimal chemotherapy and optimal surgery, respectively.

In other words, about 26% of the effect of research activity is mediated by chemotherapy and surgery.

Confidence intervals

Confidence intervals can be obtained by bootstrap resampling [6]. Simple cluster resampling preserves the dependence of patient outcomes from the same hospital (e.g. Field and Welsh [8]):

```
CSamp = function(d)
{
    s = sample(unique(d$HospID), replace=TRUE)
    return(do.call('rbind', lapply(s, function(x) d[d$HospID == x, ])))
}
```

Cluster-aware bootstrap confidence intervals are then easily obtained by repeated evaluation of `doEffectDecomp` for different bootstrap resamples.

```
HRs = replicate(10000, doEffectDecomp(CSamp(d)))
apply(HRs, 1, quantile, c(0.025, 0.975))
```

The last command produces the following output:

```
      TrialTRUE  MedChemTRUE  MedSurgTRUE   Age5  ECOG0/1  Ascit>500 ml
2.5%      0.474        0.837        0.841  1.140    1.380         1.250
97.5%     0.917        1.013        1.019  1.360    3.280         2.670

     ComorbTRUE  HistoSerous     GradeG3/4     TE       DE       IE       PM
2.5%      1.030        0.850        0.760  0.411    0.474    0.751    0.033
97.5%     2.010        2.060        1.500  0.801    0.917    0.984    0.686
```

The 95% confidence intervals for the mediator hazard ratios are [0.837; 1.013] and [0.841; 1.019] for chemotherapy and surgery, respectively. The 95% confidence interval for the indirect HR = 0.868 (i.e. mediated effect) is [0.751; 0.984]. The proportion mediated is within 3.3% and 68.6%.

Sensitivity analysis 1: One binary mediator

The multiple pathway framework [7] assumes that that the two mediators are fulfilled independently of each other, as well as that the two mediators operate separately of each other. Because it is generally difficult to test such assumptions within the same data set, we provide two sensitivity analyses to assess the robustness of the results.

In the first analysis, a single binary mediator is used that reflects optimal adherence to treatment guidelines in the sense that both the chemotherapy and surgery are considered optimal:

```
d$Opti = d$Chem & d$Surg # Single binary mediator for optimal treatment

MOpti = geeglm(Opti ~ Trial + Age5 + ECOG + Ascit + Comorb + Histo + Grade,
    family=binomial(), data=d, id=d$HospID)
summary(MOpti)
```

There is again a significant effect of research activity on the aggregate mediator as shown by logistic regression, with an odds ratio of exp(0.566) = 1.76 in favor of research-active hospitals.

```
              Estimate  Std.err  Wald Pr(>|W|)
(Intercept)     3.8963   0.8786 19.66  9.2e-06 ***
TrialTRUE       0.5655   0.2853  3.93    0.047 *
(output cropped)
```

Because there is only mediator, effect decomposition is simplified compared to the main analysis, with only one counterfactual duplication of the data:

```
doEffectDecomp = function(d)
{
    # Step 1: Replicate exposure variable, predict mediator
    d$TrialTemp = d$Trial
    MOpti = glm(Opti ~ TrialTemp + Age5 + ECOG + Ascit + Comorb + Histo +
        Grade, family=binomial(), data=d)

    # Step 2: Replicate data with different exposures for the mediator
    d1 = d2 = d
    d1$Med = d1$Trial
    d2$Med = !d2$Trial
    newd = rbind(d1, d2)

    # Step 3: Compute weights for the mediator
    newd$TrialTemp = newd$Trial
    w = predict(MOpti, newdata=newd, type='response')
    direct = ifelse(newd$Opti, w, 1-w)
    newd$TrialTemp = newd$Med
    w = predict(MOpti, newdata=newd, type='response')
    indirect = ifelse(newd$Opti, w, 1-w)
    newd$W = indirect/direct

    # Step 4: Weighted Cox Model
    cox = coxph(Surv(OS, Status) ~ Trial + Med + Age5 + ECOG + Ascit +
        Comorb + Histo + Grade, weight=W, data=newd)

    # Return value: Estimates for total, direct, indirect effect
    TE = exp(sum(coef(cox)[c('TrialTRUE', 'MedTRUE')]))
    DE = exp(unname(coef(cox)['TrialTRUE']))
    IE = exp(sum(coef(cox)['MedTRUE']))
    PM = log(IE) / log(TE)

    return(c(exp(coef(cox)), TE=TE, DE=DE, IE=IE, PM=PM))
}
```

Point estimates are again obtained by directly invoking `doEffectDecomp`:

```
(...)        TE            DE           IE           PM
(...)     0.580         0.635        0.914        0.166
```

The code for the bootstrap confidence interval is unchanged, the result is as follows:

```
            TE       DE       IE          PM
2.5%     0.416    0.451    0.817    0.000894
97.5%    0.806    0.886    0.999    0.514792
```

Hence, the total HR = 0.580 (95% CI: 0.416 to 0.806) decomposes into a direct HR = 0.635 (CI: 0.451 to 0.886) and an indirect HR = 0.914 (CI: 0.817 to 0.999). The effect estimates and confidence intervals, thus, roughly correspond to those of the primary analysis. Because the two mediators have been collapsed to one single mediator, the indirect effect and the mediated proportion are attenuated and a greater proportion of the trial participation effect operates via the direct path.

Sensitivity analysis 2: One mediator with three categories

In a second sensitivity analysis, we counted the number of fulfilled criteria, that is, the mediator was again a single variable indicating whether none (neither chemotherapy nor surgery), one (optimal chemotherapy or optimal surgery), or both criteria for treatment adherence (optimal chemotherapy and optimal surgery) were met.

```
d$NOpti = as.ordered(d$Chem + d$Surg)       # Mediator for 0, 1, 2 criteria

library(VGAM) # Needs to be downloaded from CRAN
MNOpti = vglm(NOpti ~ Trial + Age5 + ECOG + Ascit + Comorb + Histo + Grade,
    family=propodds(), data=d)
exp(coef(MNOpti))
```

The effect of research activity on `NOpti` is studied using proportional odds ordinal regression [9], adjusting again for the known baseline confounders.

```
(Intercept):1 (Intercept):2     TrialTRUE
      725.480        65.155         1.861
```

The odds ratio for trial participation is 1.86, indicating again better adherence to treatment guidelines in the research-active hospitals.

The effect decomposition now determines the mediation effects using fixed counterfactual exposure levels in Step 2. In Step 3 the weights are determined by selecting the column of the counterfactual prediction matrix that matches to the actually observed mediator (the ordered factor is automatically coerced to a numeric column index).

```
doEffectDecomp = function(d)
{
    # Step 1: Replicate exposure variable, predict mediator
    d$TrialTemp = d$Trial
    MNOpti = vglm(NOpti ~ TrialTemp + Age5 + ECOG + Ascit + Comorb +
        Histo + Grade, family=propodds(), data=d)

    # Step 2: Replicate data with different exposures
    d1 = d2 = d
    d1$TrialStar = TRUE
    d2$TrialStar = FALSE
    newd = rbind(d1, d2)

    # Step 3: Compute weights for the mediator
    newd$TrialTemp = newd$Trial
    direct = predict(MNOpti, newdata=newd,
        type='response')[cbind(1:nrow(newd), newd$NOpti)]
    newd$TrialTemp = newd$TrialStar
    indirect = predict(MNOpti, newdata=newd,
        type='response')[cbind(1:nrow(newd), newd$NOpti)]
    newd$W = indirect/direct

    # Step 4: Weighted Cox Model
    cox = coxph(Surv(OS, Status) ~ Trial + TrialStar + Age5 + ECOG +
        Ascit + Comorb + Histo + Grade, weight=W, data=newd)
```

```
    # Return value: Estimates for total, direct, indirect effect
    TE = exp(sum(coef(cox)[c('TrialTRUE', 'TrialStarTRUE')]))
    DE = exp(unname(coef(cox)['TrialTRUE']))
    IE = exp(sum(coef(cox)['TrialStarTRUE']))
    PM = log(IE) / log(TE)
    return(c(exp(coef(cox)), TE=TE, DE=DE, IE=IE, PM=PM))
}
```

Point estimates and confidence intervals are again obtained by `doEffectDecomp(d)` and bootstrap resampling:

```
(...)          TE       DE       IE       PM
(...)       0.579    0.673    0.861    0.275

    (...)      TE       DE       IE       PM
2.5%        0.414    0.481    0.753    0.054
97.5%       0.805    0.923    0.973    0.674
```

The 95% confidence interval for the indirect HR = 0.861 (i.e. mediated effect) is thus [0.753; 0.973], which is very close to the two-mediator solution.

Sensitivity analysis 3: Non-linearity, interactions and misclassification

In a last sensitivity analysis, we investigated possible bias due to non-linear relationships and interactions between exposure, baseline variables and mediators, as well as possible misclassification of the mediators. As the logistic regression of mediators shows, there is a possible non-linear relationship between age and the mediators as well as some spurious interaction between age and exposure:

```
anova(geeglm(Chem ~ Trial * (Age5 + ECOG + Ascit + Comorb + Histo + Grade)
    + I(Age5^2), family=binomial(), data=d, id=d$HospID))

anova(geeglm(Surg ~ Trial * (Age5 + ECOG + Ascit + Comorb + Histo + Grade)
    + I(Age5^2), family=binomial(), data=d, id=d$HospID))
```

```
Response: Chem

             Df   X2 P(>|Chi|)
Trial         1  3.6    0.0583 .
Age5          1 39.9   2.6e-10 ***
ECOG          1 19.0   1.3e-05 ***
Ascit         1  0.7    0.3880
Comorb        1  4.8    0.0287 *
Histo         1  1.3    0.2585
Grade         1  0.0    0.8272
I(Age5^2)     1 24.4   7.7e-07 ***
Trial:Age5    1  7.1    0.0078 **
Trial:ECOG    1  2.7    0.0981 .
Trial:Ascit   1  2.6    0.1038
Trial:Comorb  1  0.2    0.6525
Trial:Histo   1  0.6    0.4494
Trial:Grade   1  0.0    0.9861
```

```
Response: Surg

             Df    X2 P(>|Chi|)
Trial         1  3.59   0.05803 .
Age5          1 25.43   4.6e-07 ***
ECOG          1 11.05   0.00089 ***
Ascit         1 13.37   0.00026 ***
Comorb        1  2.10   0.14740
Histo         1  0.74   0.39065
Grade         1  0.02   0.89247
I(Age5^2)     1  0.06   0.80419
Trial:Age5    1  1.06   0.30289
Trial:ECOG    1  1.18   0.27756
Trial:Ascit   1  4.19   0.04075 *
Trial:Comorb  1  0.30   0.58570
Trial:Histo   1  0.14   0.71246
Trial:Grade   1  1.29   0.25552
```

We therefore included Age5² and the interaction between Age and Trial participation in the logistic regression for the effect decomposition:

```
doEffectDecomp = function(d)
{
    # Step 1: Replicate exposure variable, predict mediators
    d$TrialTemp = d$Trial

    MChem = glm(Chem ~ TrialTemp * Age5 + I(Age5^2) + ECOG + Ascit + Comorb
        + Histo + Grade, family=binomial(), data=d)

    MSurg = glm(Surg ~ TrialTemp * Age5 + I(Age5^2) + ECOG + Ascit + Comorb
        + Histo + Grade, family=binomial(), data=d)
...
```

The result is only slightly affected by the inclusion of Age5^2 and the interaction between Age and Trial.

```
 TrialTRUE  MedChemTRUE  MedSurgTRUE     Age5  ECOG>1      Ascit>500 ml
     0.644        0.960        0.919    1.213   2.186             1.747

ComorbTRUE  HistoSerous    GradeG3/4       TE      DE        IE       PM
     1.391        1.254        1.134    0.568   0.644     0.882    0.222
```

Nonlinearities and interactions can lead to unexpected bias in case of misclassification. We therefore ran an additional sensitivity analysis with 15% misclassification at random in the two mediators:

```
doEffectDecomp = function(d)
{
    # Step 1: Replicate exposure variable, predict mediators
    d$TrialTemp = d$Trial

    d$Chem <- ifelse(runif(nrow(d)) < 0.85, d$Chem, !d$Chem)
    d$Surg <- ifelse(runif(nrow(d)) < 0.85, d$Surg, !d$Surg)

    MChem = glm(Chem ~ TrialTemp * Age5 + I(Age5^2) + ECOG + Ascit + Comorb
        + Histo + Grade, family=binomial(), data=d)
...
```

The results (average of 500 simulated point estimates) indicate that in the particular data, misclassification attenuates the effect estimate for the mediated effect:

```
 TrialTRUE  MedChemTRUE  MedSurgTRUE    Age5   ECOG>1        Ascit>500 ml
     0.608        0.989        0.965   1.246    2.155               1.840

ComorbTRUE  HistoSerous    GradeG3/4      TE       DE       IE       PM
     1.485        1.334        1.111   0.580    0.608    0.954    0.115
```

References

1. du Bois A, Rochon J, Lamparter C, Pfisterer J: **Pattern of care and impact of participation in clinical studies on the outcome in ovarian cancer.** *Int J Gynecol Cancer 2005*, **15:**183–191.
2. Rochon J, du Bois A: **Clinical research in epithelial ovarian cancer and patients' outcome.** *Ann Oncol* 2011, **22**(Suppl 7):vii16–vii19.
3. R Core Team: **R: A language and environment for statistical computing.** R Foundation for Statistical Computing, Vienna, Austria, 2013.
4. Halekoh U, Højsgaard S, Yan J: **The R package geepack for generalized estimating equations.** *J Stat Soft* 2006, **15:**2.
5. Therneau T: **A Package for Survival Analysis in S**. R package version 2.37-4, http://CRAN.R-project.org/package=survival; 2013.
6. Lange T, Vansteelandt S, Bekaert M: **A simple unified approach for estimating natural direct and indirect effects.** *Am J Epidemiol* 2012, **176:**190–195.
7. Lange T, Rasmussen M, Thygesen LC: **Assessing natural direct and indirect effects through multiple pathways.** *Am J Epidemiol* 2014, **179:**513–518.
8. Field CA, Welsh AH: **Bootstrapping clustered data.** *J Roy Statist Soc Ser B Methodological.* 2007, **69:**369–390.
9. Yee TW: **The VGAM Package for Categorical Data Analysis**. *J Stat Soft* 2010, **32**:10.
